# Purpura Hemorrhagica Treated with Antistreptococcus Serum; Recovery

**Published:** 1898-06

**Authors:** L. R. Webber


Dr. L. R. Webber, Rochester, N. Y., reports a case of influenza in a bay mare, discharged some two days before as convalescent, showing on April 26th facial, nasal, tracheal, breast, thigh, and very marked swellings of the forelimbs, especially of the knee-joints; anorexia, pulse 56, and temperature 105°. Diagnosis: Purpura hemorrhagica; recommended antistreptococcus serum. Placed animal in infirmary on the 26th, clipped hair from neck and shoulders, and washed the parts with 1 : 500 bichloride solution, then injected 30 c.c. in three doses of 10 c.c. each at three points. At night she ate some oats and bran. On the 27th the pulse was 48, temperature 103°, swellings diminished; repeated same amount of serum. On the 28th the pulse was 42, temperature 100°, swellings much reduced, appetite good; repeated the same amount, 30 c.c. On the 29th the pulse was 40, temperature 100°; swellings of the body had disappeared. Having purchased 100 c.c., gave the remaining 10 c.c.; vegetable and mineral tonics were given along. On May 1st the swellings began to return, appetite diminished, mucous membranes became more discolored, and I obtained 100 c.c. of serum and gave 30 c.c. the first day, 20 c.c. the next two days, with a continued abatement of all the symptoms. On May 11th the swellings had all disappeared, appetite good, pulse and temperature normal, and only a slightly injected condition of nasal mucous membrane. In all 170 c.c were given.

